# Sprint interval exercise versus continuous moderate intensity exercise: acute effects on tissue oxygenation, blood pressure and enjoyment in 18–30 year old inactive men

**DOI:** 10.7717/peerj.7077

**Published:** 2019-06-07

**Authors:** Yuri Kriel, Christopher D. Askew, Colin Solomon

**Affiliations:** School of Health and Sports Sciences, University of the Sunshine Coast, Sippy Downs, Australia

**Keywords:** HIIT, SIT, CMIE, NIRS, Blood pressure, Enjoyment, Sprint interval training, Continuous moderate intensity exercise, Inactive

## Abstract

**Background:**

Sprint interval training (SIT) can be as effective, or more effective, than continuous moderate intensity exercise (CMIE) for improving a primary risk factor for cardiometabolic disease, low cardiorespiratory fitness (CRF). However, there has been no direct comparison in inactive individuals, of the acute effects of a session of SIT with a work-matched session of CMIE on local oxygen utilisation, which is a primary stimulus for increasing CRF. Furthermore, post-exercise blood pressure (BP) and enjoyment, if symptomatic and low, respectively, have implications for safety and adherence to exercise and have not been compared between these specific conditions. It was hypothesised that in young inactive men, local oxygen utilisation would be higher, while post-exercise BP and enjoyment would be lower for SIT, when compared to CMIE.

**Methods:**

A total of 11 inactive men (mean ± SD; age 23 ± 4 years) completed a maximal ramp-incremental exercise test followed by two experiment conditions: (1) SIT and (2) work-matched CMIE on a cycle ergometer on separate days. Deoxygenated haemoglobin (∆HHb) in the pre-frontal cortex (FH), gastrocnemius (GN), left vastus lateralis (LVL) and the right vastus lateralis (RVL) muscles, systemic oxygen utilisation (VO_2_), systolic (SBP) and diastolic (DBP) blood pressure and physical activity enjoyment scale (PACES) were measured during the experiment conditions.

**Results:**

During SIT, compared to CMIE, ∆HHb in FH (*p* = 0.016) and GN (*p* = 0.001) was higher, while PACES (*p* = 0.032) and DBP (*p* = 0.043) were lower. No differences in SBP and ∆HHb in LVL and RVL were found between conditions.

**Conclusions:**

In young inactive men, higher levels of physiological stress occurred during SIT, which potentially contributed to lower levels of post-exercise DBP and enjoyment, when compared to CMIE.

## Introduction

Physical activity recommendations for health include accumulating 150–300 min of moderate intensity exercise each week (Egger et al., 2001; Riebe et al., 2018). A frequently cited reason for non-compliance with these recommendations, in the general population (Strazdins et al., 2011) and in young men (Ashton et al., 2017) is insufficient time. High intensity interval training (HIIT), including time-efficient sprint interval training (SIT), has been shown to be as effective (Macpherson et al., 2011) or more effective (Tjønna et al., 2008) than longer duration continuous moderate intensity exercise (CMIE) at improving specific cardiovascular disease risk factors, such as low cardiorespiratory fitness (CRF) (Kessler, Sisson & Short, 2012; Weston, Wisloff & Coombes, 2014a). Therefore, SIT appears to represent a potent stimulus for increasing CRF and attaining associated health gains (Gillen & Gibala, 2014, 2018). However, the local factors contributing to the greater CRF improvements shown during SIT are not completely understood. A more comprehensive understanding of the response at the local tissue level to the stimulus of SIT, compared to CMIE, would inform the choice between these exercise formats for the efficient achievement of health related outcomes linked to increases in CRF.

Increased CRF, as a result of SIT and CMIE, has been attributed partly to increases in mitochondrial biogenesis, content and function (De Strijcker et al., 2018; Jacobs et al., 2013; Russell et al., 2014; Skelly & Gillen, 2018; Skelly et al., 2018). Whilst the exact mechanisms underlying the increases in mitochondria are yet to be fully elucidated, it is possible that a larger increase in tissue oxygen utilisation during a session of SIT, when compared to a session of CMIE (Bailey et al., 2009), provides a greater stimulus/metabolic stress leading to the enhanced mitochondrial and associated aerobic adaptations (De Strijcker et al., 2018; Fiorenza et al., 2018). Site specific oxygen utilisation at the local tissue level can be measured using near infrared spectroscopy (NIRS). NIRS is a non-invasive method for the measurement of the change in concentration of oxyhaemoglobin (∆O_2_Hb) (oxygen availability) and deoxygenated haemoglobin (∆HHb) (oxygen utilisation). Oxygen utilisation has been described during SIT in active individuals at a single muscle site (Jones, Hamilton & Cooper, 2015) and in the pre-frontal cortex (Monroe et al., 2016; Smith & Billaut, 2010), and is increased when compared to pre-exercise values (Dupont et al., 2007). Comparisons of locomotor muscle oxygenation have found no difference between SIT and CMIE in active individuals (Bailey et al., 2009; McKay, Paterson & Kowalchuk, 2009). However, these comparisons utilised steady state or step transition exercise tests before and after a period of exercise training, not an acute work-matched comparison. Given that SIT is proposed as an exercise training format to engage inactive individuals who are potentially time-poor (Astorino & Thum, 2016; Del Vecchio et al., 2015; Jung, Little & Batterham, 2016), and thereby improve risk factors including low CRF in a time-efficient manner, there is a need to better understand the acute local oxygen utilisation responses to SIT versus CMIE in this population on a matched ‘unit-by unit’ basis. By investigating the oxygen utilisation responses in inactive individuals at multiple tissue sites in response to a session of work-matched SIT and CMIE, the primary aim of this study is to define the extent of the disparity in oxygen utilisation between SIT and CMIE, and thereby provide an insight into the contribution of tissue oxygen utilisation as a potential stimulus for improvements in CRF and potentially other health parameters specific to the brain (Lucas et al., 2015).

The safety of SIT in inactive individuals has been contended (Halle, 2013; Keteyian, 2012; Levinger et al., 2015; Rognmo et al., 2012, 2013; Weston, Wisloff & Coombes, 2014a), partly due to the fact that symptomatic post-exercise hypotension (PEH) has been linked to high intensity exercise (Mundel et al., 2015), sedentary status (MacDonald, 2002) and low CRF (Dujic et al., 2006). Additionally, young adults are heavily represented in the symptomatic PEH literature (Krediet et al., 2004). It is therefore plausible to expect symptomatic hypotensive responses post-SIT, in young inactive individuals. A secondary aim of this study is therefore to compare blood pressure (BP) responses post-SIT and post-CMIE to determine if, for the same amount of work, SIT elicits greater reductions in post-exercise BP and furthermore, if reductions in BP coincide with symptoms that may influence the safety and potentially also the enjoyment of SIT.

While SIT is a time-efficient mode of exercise shown to improve CRF (Weston et al., 2014b), its adoption by inactive individuals may be limited by the extent to which it is enjoyed. The comparison of enjoyment during HIIT, SIT and CMIE has yielded contradictory results in insufficiently active participants (Stork et al., 2017). Individuals are more likely to enjoy exercise that they perceive as moderate in intensity (Williams et al., 2008), however, no difference in enjoyment levels has been found between sessions of HIIT, SIT and CMIE (Jung, Bourne & Little, 2014; Oliveira et al., 2013; Olney et al., 2018; Stork, Gibala & Ginis, 2018). These previous comparisons have not controlled for the total work performed during the exercise sessions. Differing amounts of work across the sessions could influence the physiological demands and enjoyment of the exercise format, especially in untrained or inactive individuals, and therefore obscure findings. Comparing enjoyment levels between a session of SIT and a session of work-matched CMIE is necessary to determine if SIT can effectively take the place of CMIE as a primary strategy for improving CRF and the prevention of chronic disease (Coyle, 2005; Kessler, Sisson & Short, 2012; Whyte, Gill & Cathcart, 2010) in a population with low intrinsic motivation to exercise (Aaltonen et al., 2014; Ashton et al., 2017).

It was hypothesised that in young inactive men, Δ[HHb] would be higher at all sites while post-exercise BP and enjoyment would be lower for a session of SIT, compared to a work-matched session of CMIE.

## Materials and Methods

### Ethics statement

This research study was approved by the human research ethics committee of the University of the Sunshine Coast (S/13/472). All participants received a research project information sheet before providing written informed consent.

### Experiment design

**Figure 1:**
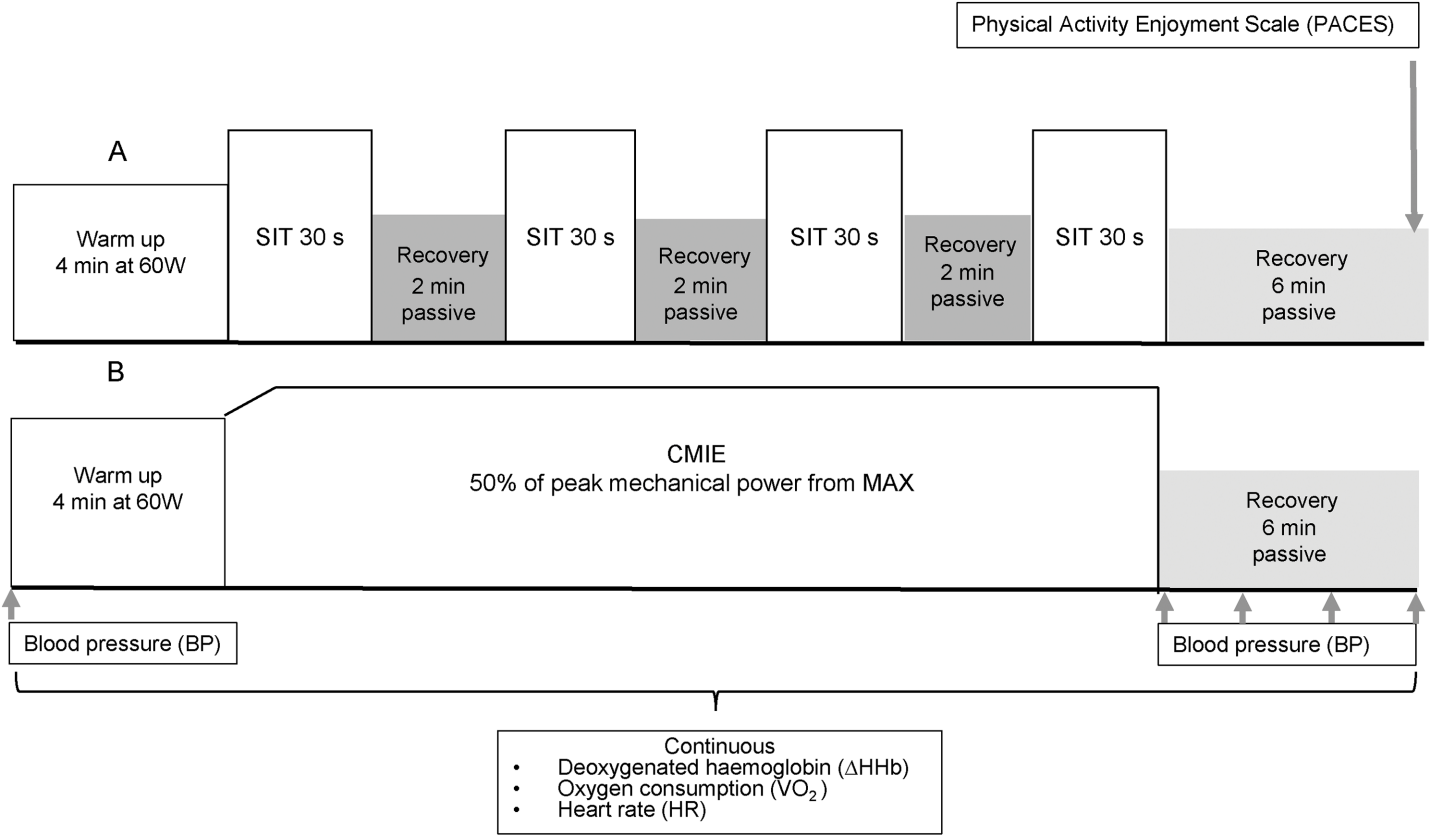
The format and timing of measurements of the two experiment conditions. (A) Sprint interval training (SIT) and (B) Continuous moderate intensity exercise (CMIE).

This study consisted of a maximal ramp-incremental exercise test (MAX) followed by two experiment conditions performed on a cycle ergometer in the following order: (1) SIT and (2) CMIE matched for the mechanical work performed during SIT. All sessions were separated by 3 to 7 days to minimise the influence of potential carry-over effects or confounding variables. The ∆HHb in the pre-frontal cortex (FH), gastrocnemius (GN), left vastus lateralis (LVL) and the right vastus lateralis (RVL) muscles, VO_2_, heart rate (HR), post-exercise BP, and enjoyment via the physical activity enjoyment scale (PACES) were measured during the sessions. The format of the experiment conditions and the timing of measurements are illustrated in Fig. 1.

### Participants

The participant group consisted of 11 men who met the inclusion criteria of being aged 18–30 years; currently completing less than 150 min of moderate intensity or 75 min of vigorous intensity activity per week; reporting no cardiovascular and metabolic disease; taking no medications; having no known orthopaedic or other health related issues that would be made worse by participation in, or inhibit completion of, the study. Descriptive characteristics of the participants are in Table 1.

**Table 1:** Participant characteristics.

Height (cm)	179 ± 8
Weight (kg)	78 ± 7
Age (year)	23 ± 4
Reported weekly physical activity time (min)	31 ± 33
VO_2peak_ (ml.kg.^−1^min^−1^) during MAX	40.7 ± 4.3
Peak heart rate (bpm) during MAX	182 ± 15
Peak mechanical power (W) during MAX	255 ± 30
Peak systolic blood pressure (mmHg) during MAX	187 ± 21
Peak diastolic blood pressure (mmHg) during MAX	80 ± 7
Left vastus lateralis skinfold (mm)	10.4 ± 2.9
Right vastus lateralis skinfold (mm)	11.8 ± 2.8
Gastrocnemius skinfold (mm)	11.3 ± 2.9
FVC (L)	5.74 ± 0.86
FVC % pred (%)	108 ± 12
FEV_1_ (L)	4.82 ± 0.78
FEV_1_ % pred (%)	107 ± 11

**Note:**

VO_2peak_, Peak maximal oxygen uptake; FVC, Forced vital capacity; FEV1, Forced expiratory volume in 1 s. Data are (mean ± SD) (*n* = 11).

### Procedures and equipment

#### Screening procedures

At the initial session, participants completed risk screening questionnaires, a physical activity log and characteristics including resting pulmonary function and adipose tissue thickness were measured (Table 1), as previously described (Kriel, Askew & Solomon, 2018; Kriel et al., 2016). Specifically, the physical activity log ensured that participants’ activity levels during the previous 3 months were within the definition of inactive for the purposes of this study (an individual not achieving the current minimal recommendations of 150 min of moderate or 75 min of vigorous intensity activity per week to gain health benefits) (Barnes et al., 2012). Additionally, participants reported no involvement in HIIT during the previous 3 months. Participants were asked to refrain from performing any exercise in the 24 h preceding each session and to not ingest any caffeine, alcohol or a large meal in the 4 h preceding each session (Goldstein et al., 2010). It was confirmed at each session that participants were appropriately fed and hydrated (Convertino et al., 1996; Garzon & Mohr, 2014).

#### Exercise conditions

Prior to MAX, participants were familiarised with the exercise protocols, the Velotron cycle ergometer (Racermate, Seattle, WA, USA) and the process of maintaining a constant cadence.

As previously described (Kriel et al., 2016), each exercise session began with an initial 3 min baseline data collection period during which the participant remained stationary on the cycle ergometer. The baseline period was followed by a 4 min warm up period, which consisted of cycling against a fixed resistance of 60 W at a cadence of 60 revolutions per minute. Participants were instructed to remain seated throughout each session to reduce movement artefact in the NIRS signal and to allow for consistency in muscular recruitment patterns and hence power data. During MAX, the warm up was followed by a ramp-incremental protocol, during which the resistance was increased by 20 W every minute until volitional cessation.

During SIT, as previously described (Kriel, Askew & Solomon, 2018; Kriel et al., 2016), the warm up was followed by four 30 s bouts of sprint exercise, with 2 min passive recovery periods separating each bout. Each participant was asked to increase cadence to a maximum during a 5 s period immediately preceding each bout. Power output during the Wingate style SIT bouts was determined by participant effort against the resistance (0.075 kg per kilogram body weight) applied to the flywheel of the ergometer. The SIT format and timings were adapted from protocols used previously in active and sedentary populations (Buchheit et al., 2012; Burgomaster et al., 2008, 2005; Dupont et al., 2007; Gibala & McGee, 2008; Stork et al., 2017). Participants were instructed to give a maximal effort from the beginning of each bout, using the prompt to ‘go as hard as you can’. Participants were verbally encouraged using standardised phrases during all bouts of exercise to promote a maximal effort. During the 2 min passive recovery periods between bouts, participants were instructed to sit as still as possible to reduce movement artefact in the NIRS data and to ensure that no mechanical work was performed.

During CMIE, the warm up was followed by moderate intensity exercise completed at 50% of the peak power output achieved during MAX. The CMIE condition was matched to the mechanical work performed during the SIT condition. Therefore, the length of the CMIE condition was specific to each participant (range 5:33–7:38 min). No significant differences in mechanical work were found between the experiment conditions (SIT 47.09 ± 5 kJ, CMIE 45.4 ± 5 kJ). The slight differences in mechanical work between conditions were due to inactive participants having difficulty holding a constant power output during CMIE. At the completion of all exercise sessions, there was a 6 min passive recovery period, during which measurements continued as illustrated in Fig 1.

#### Tissue oxygenation

Changes in local tissue oxygenation were measured continuously during rest, exercise and recovery, as described previously (Kriel, Askew & Solomon, 2018; Kriel et al., 2016). Specifically, the changes in the relative concentration of O_2_Hb (∆[O_2_Hb]) and HHb (∆[HHb]) were measured using a NIRS system (3 × PortaMon and 1 × Portalite devices; Artinis Medical Systems BV, Zetten, Netherlands). The PortaMon devices were placed over the muscle belly of three locomotor muscles: the LVL, the RVL and the left GN and the Portalite optode was placed on the area of the forehead over the pre-frontal cortex (FH). Between test reliability of the HHb data from the NIRS system was examined prior to this study, providing acceptable absolute reliability values of the baseline data for each site (Typical Error: LVL = 0.4 μM, GN = 0.8 μM, FH = 0.6 μM) (Kriel et al., 2016). For all testing, the same device was used at the same measurement site, for all participants.

Only Δ[HHb] values are presented, as discussed previously (Kriel, Askew & Solomon, 2018; Kriel et al., 2016). Specifically, the ∆[HHb] data are potentially unaffected by changes in perfusion, blood volume or arterial haemoglobin concentration (Adami et al., 2015; Jones et al., 2009; Wang et al., 2006). The ∆[O_2_Hb] data are affected by muscular compression and changes in blood flow and volume (Grassi et al., 2003), especially during the rapid and substantial changes in these variables associated with SIT bouts (Buchheit et al., 2009). However, despite precautions, gross movement artefact was present in NIRS data collected at the LVL, RVL and GN sites during the passive recovery periods of the SIT sessions, ascribed to a low signal to noise ratio during the recovery periods. Therefore, the passive recovery period data were not included in analysis. Similar technical difficulties in NIRS data collection, leading to data exclusion, has occurred in other research (Mekari et al., 2015).

### Systemic oxygen consumption, heart rate and mechanical power

As described previously (Kriel, Askew & Solomon, 2018), systemic oxygen consumption (VO_2_) data were collected using a respiratory gas analysis open circuit spirometry system (Parvo Medics, Sandy, UT, USA) and a standard gas collection mouthpiece (Hans Rudolph, Shawnee Mission, KS, USA). To quantify HR response, a HR monitor (RS400; Polar Electro, Kempele, Finland) was used. To quantify mechanical power a crank-based power meter (SRM Science, Schoberer Rad Meßtechnik, Julich, Germany) was used. VO_2_ and HR were collected continuously during rest and exercise periods and mechanical power was collected continuously during exercise periods. Standardised calibration and methods were used for these variables (Macfarlane & Wu, 2013).

### Blood pressure

Systolic (SBP) and diastolic (DBP) blood pressure responses were measured at rest, immediately upon cessation of exercise (Post Ex) and every 2 min during the 6 min recovery period (Rec 2, Rec 4 and Rec 6) using a manual blood pressure cuff, aneroid and stethoscope (Welch Allyn, Skaneateles Falls, NY, USA).

### Physical activity enjoyment scale

As described previously (Kriel, Askew & Solomon, 2018), participants completed the PACES questionnaire within 5 min of completing SIT and CMIE to determine enjoyment level in response to each condition. The PACES consists of 18 items on a 7-point bipolar scale. A minimum total score of 18 and a maximal total score of 126 is possible.

### Data calculation

All NIRS data were collected at a frequency of 10 Hz and smoothed using a 10-point moving average before being averaged to 1 s periods. Due to the HHb data being a measure of change from an assigned baseline zero value, the HHb data were expressed as units of change (μM) from the mean value of the 30 s of baseline data preceding the start of exercise (∆[HHb]). The VO_2_ data were averaged over 5 s periods and HR and power data were averaged at 1 s intervals. The NIRS, HR, VO_2_ and power data were then time aligned and the time periods of data corresponding to the exercise periods of each condition identified. Mean values for each exercise period were then calculated for all dependant variables for each condition, providing a single value per condition for statistical analysis. The passive recovery periods during the HIIT session were not included in the calculation of mean values. The SBP, DBP and PACES data provided a single value per measurement time for statistical analysis.

### Statistics

Statistical tests were performed using the IBM SPSS Statistics (version 22; IBM Corporation, Armonk, NY, USA) program. Data were initially tested for normality of distribution using the Shapiro–Wilk test. A two factor, repeated-measures analysis of variance was used to evaluate the main and interaction effects of: condition (SIT *vs* CMIE) and site (FH, GN, LVL, RVL) on the dependant variable ∆[HHb] as well as condition and time (Rest, Post Ex, Rec 2, Rec 4, Rec 6) on SBP and DBP. If a significant main effect was identified, a Bonferroni’s post hoc test was used to make pair wise comparisons. A paired samples *T*-test was used to analyse the effect of condition on VO_2_, HR, mechanical power and PACES scores. All data are presented as mean ± standard deviation (SD). For all statistical analyses, a *P*-value of <0.05 was used as the level of significance. Effect size estimates are indicated using partial eta-squared values.

## Results

### Tissue oxygenation

**Figure 2:**
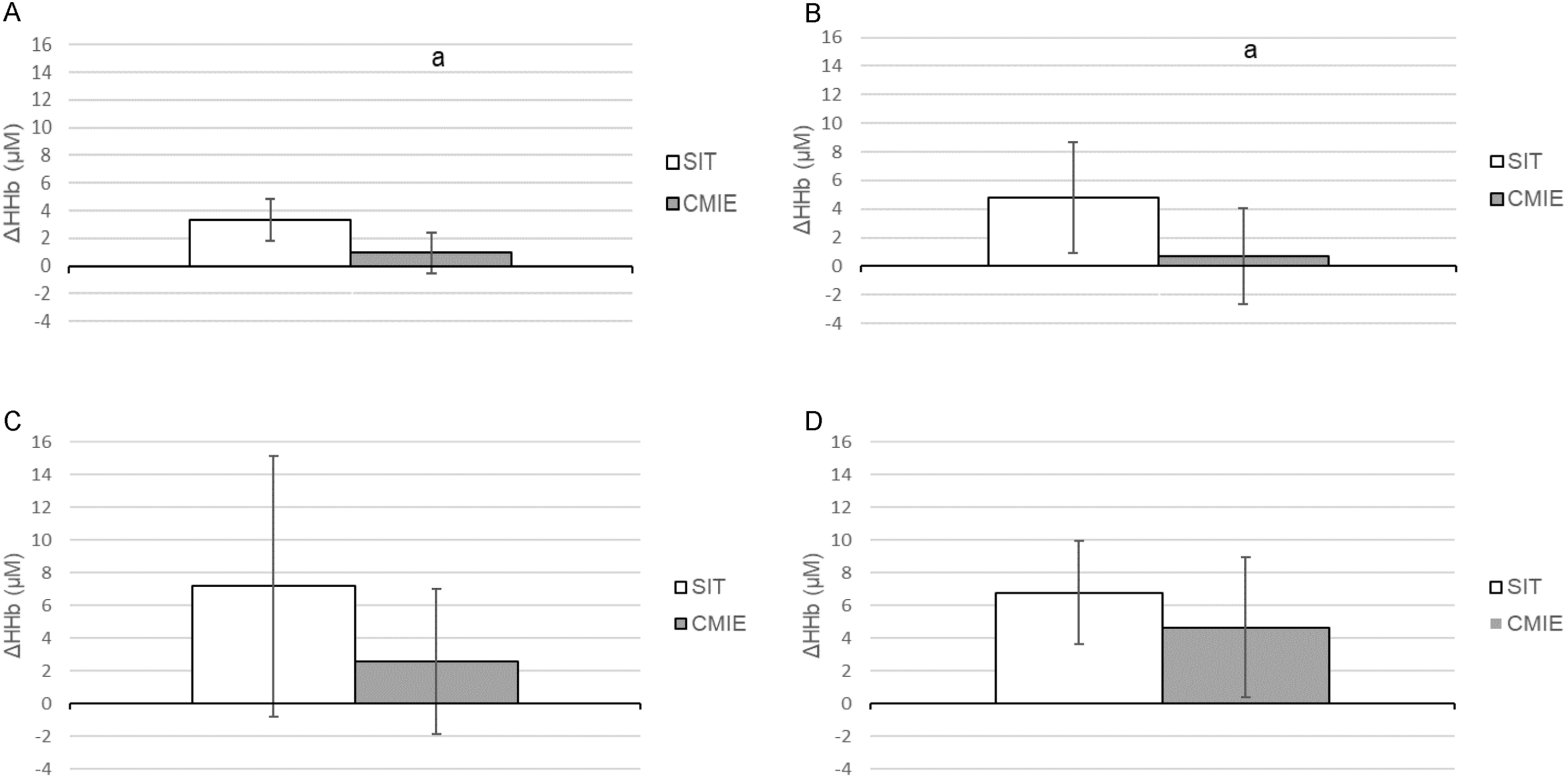
Change in deoxygenated haemoglobin (HHb) concentration during sprint interval training (SIT) and continuous moderate intensity exercise (CMIE). (A) Forehead. (B) Gastrocnemius. (C) Left vastus lateralis. (D) Right vastus lateralis. a = significantly different to SIT. Data are mean ± SD. (*p* ≤ 0.05).

For the Δ[HHb] for all sites and conditions, there was a main effect for site (*p* = 0.032, *F* = 3.387, η_p_^2^ = 0.273) and condition (*p* = 0.001, *F* = 24.135, η_p_^2^ = 0.728). For the Δ[HHb] for each condition, there was no main effect for site. For the FH and GN, Δ[HHb] was higher during SIT compared to CMIE (*p* = 0.016, *F* = 8.783, η_p_^2^ = 0.494) and (*p* = 0.001, *F* = 18.76, η_p_^2^ = 0.652) respectively (Figs. 2A and 2B). For the LVL and RVL, no significant differences were found for the Δ[HHb] between conditions (Figs. 2C and 2D).

### Systemic oxygen consumption, heart rate and mechanical power

**Figure 3:**
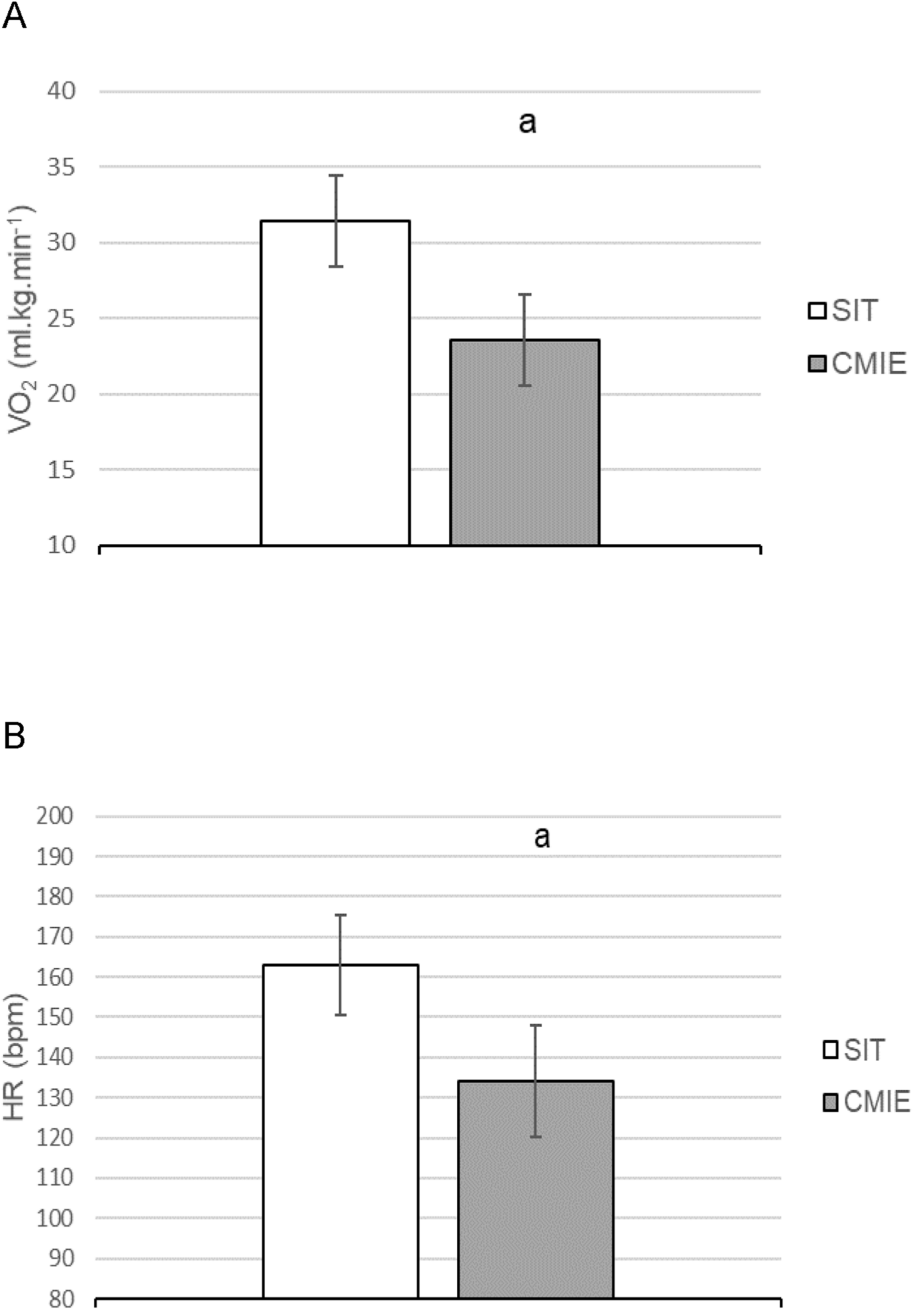
Oxygen consumption and heart rate during sprint interval training (SIT) and continuous moderate intensity exercise (CMIE). (A) Oxygen consumption (VO_2_). (B) Heart rate (HR). a = significantly different to SIT. Data are mean ± SD. (*p* ≤ 0.05).

The VO_2_ and HR were higher; (*t*(9) = 11.243, *p* < 0.001, η_p_^2^ = 0.934; *t*(10) = 9.718, *p* < 0.001, η_p_^2^ = 0.904), respectively during SIT compared to CMIE. (Figs. 3A and 3B). Mechanical power was higher; (*t*(10) = 21.811, *p* < 0.001, η_p_^2^ = 0.979) during SIT (392 ± 48 W) compared to CMIE (130 ± 15 W), as expected due to study design (the inherent differences in exercise intensity between the two conditions).

### Blood pressure

**Figure 4:**
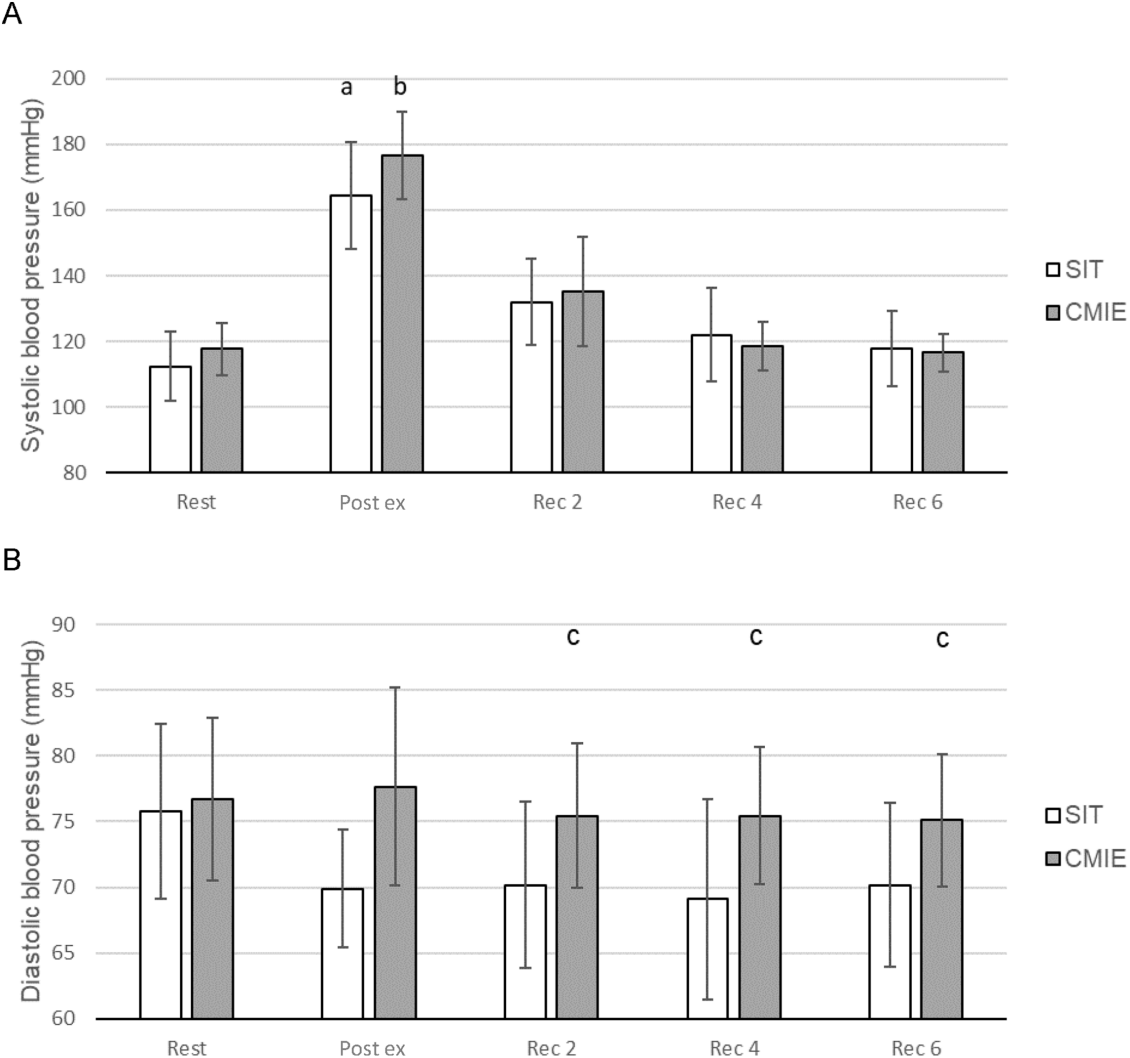
Blood pressure responses during sprint interval training (SIT) and continuous moderate intensity exercise (CMIE). (A) Systolic blood pressure (SBP). a = significantly different to SIT Rest; b = significantly different to CMIE Rest. (B) Diastolic blood pressure (DBP). c = significantly different to SIT at same time. Data are mean ± SD. (*p* ≤ 0.05).

For the post-exercise SBP there was a main effect for time (*p* < 0.001, *F* = 78.564, η_p_^2^ = 0.940). For the SBP within conditions (Fig. 4A), differences were found over time in SIT (*p* < 0.001, *F* = 47.6143, η_p_^2^ = 0.841) and CMIE (*p* < 0.001, *F* = 68.055, η_p_^2^ = 0.932), with SBP increasing significantly during exercise, when compared to resting levels, and returning to baseline during recovery. No significant differences were found between conditions.

For the post-exercise DBP, there was a main effect for condition (*p* = 0.043, *F* = 7.231, η_p_^2^ = 0.591) and time (*p* < 0.001, *F* = 8.348, η_p_^2^ = 0.625). There was a significant condition × time interaction (*p* = 0.042, *F* = 2.386, η_p_^2^ = 0.323). For the DBP for each measurement time (Fig. 4B), differences were found between conditions for Rec 2 (*p* = 0.038, *F* = 5.682, η_p_^2^ = 0.362), Rec 4 (*p* = 0.017, *F* = 8.091, η_p_^2^ = 0.447) and Rec 6 (*p* = 0.031, *F* = 6.328, η_p_^2^ =0.388).

### Physical activity enjoyment scale

**Figure 5:**
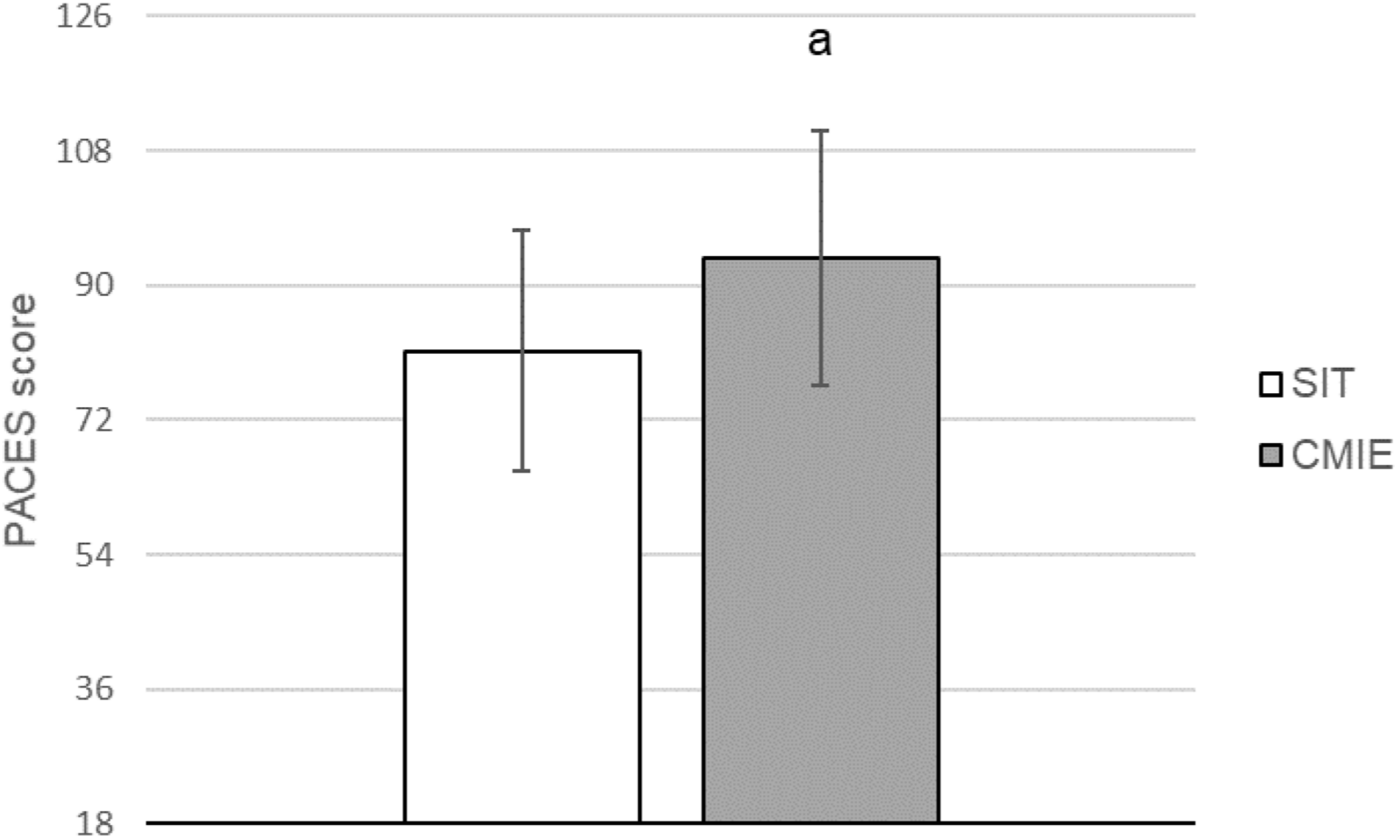
Physical activity enjoyment scale for sprint interval training (SIT) and continuous moderate intensity exercise (CMIE). Physical activity enjoyment scale (PACES). a = significantly different to SIT. Data are mean ± SD. (*p* ≤ 0.05).

The PACES score was higher for CMIE compared to SIT; (*t*(10) = −2.482, *p* = 0.032, η_p_^2^ = 0.381) (Fig. 5).

## Discussion

The aim of this study was to compare local oxygen utilisation (Δ[HHb]), post-exercise BP and exercise enjoyment (PACES) between a session of SIT and a work-matched session of CMIE, in young inactive men.

In support of our hypotheses, a higher Δ[HHb] at the FH and GN sites, a lower PACES score and a lower post-exercise DBP were found for SIT, compared to CMIE. In contrast to our hypotheses, no significant differences were found in Δ[HHb] at the LVL and RVL sites and in SBP when comparing SIT to CMIE.

### Tissue oxygenation

This study was novel in that Δ[HHb] was measured simultaneously in three skeletal muscle sites and the pre-frontal cortex in inactive men during a session of SIT and a work-matched session of CMIE.

The finding of no difference between conditions in Δ[HHb] at the LVL and RVL sites was contrary to the hypothesis of an increased oxygen utilisation during SIT in these large locomotor muscles, when compared to CMIE. This result is inconsistent with the significantly higher systemic oxygen utilisation (indicated by the higher VO_2_ and HR during SIT when compared to CMIE). However, at higher exercise intensities, increases in oxygen supply (due to increased demand) are not wholly distributed to large locomotor muscles such as the VL. The distribution of the increased systemic oxygen supply to areas other than the locomotor muscles is proposed, due to factors including the mechanical constraints of heavy contractions in the locomotor muscles during SIT and the need for the respiratory muscles to meet the increased work of breathing (Harms et al., 1997; Kime et al., 2013), potentially limiting the increase in oxygen supply to the LVL and RVL. Therefore, a higher VO_2_ and HR would not necessarily indicate an increased local oxygen utilisation at the site(s) of measurement.

Another potential contributing factor for this finding is the intermittent format of SIT and the typical Δ[HHb] response patterns at these large locomotor muscles: During 30 s bouts of SIT, Δ[HHb] increases from baseline. During the recovery periods of SIT, Δ[HHb] levels routinely decrease (recover) to baseline values (Kriel et al., 2016). During CMIE, Δ[HHb] typically increases continuously or increases and then plateaus until exercise cessation (McKay, Paterson & Kowalchuk, 2009) at a lower absolute level than those achieved during SIT. The responses during the SIT and CMIE sessions of this study conform to this pattern. These transient responses also confirm there was no evidence of a ceiling effect, as the maximal Δ[HHb] values achieved during CMIE were less than the maximal Δ[HHb] values achieved during SIT. Therefore, it is possible that, due to the different patterns of increase, similar mean responses were found between SIT and CMIE. However, the finding of no difference in mean Δ[HHb] between SIT and CMIE at the VL sites is relevant when examining the physiological effect of the sessions in their entirety, as it indicates that in these large locomotor muscles, the oxygen utilisation during a session of SIT is similar to that achieved during a work-matched session of CMIE.

Finally, the large degree of inter-individual variability in the Δ[HHb] response, indicated by the large SD (Figs. 2C and 2D) could have contributed to a non-significant statistical difference between SIT and CMIE at the LVL and RVL sites. Variable individual responses in HHb have been documented previously in research utilising NIRS (Boone et al., 2016). However, statistically significant differences were found at the FH and GN sites during this study and in three previous studies which published individual results with a similar sample size and/or a similar degree of variability in the Δ[HHb] response to the current research study (Jones, Hamilton & Cooper, 2015; Kriel, Askew & Solomon, 2018; Kriel et al., 2016).

In the smaller GN muscle, the increased oxygen utilisation during SIT, when compared to CMIE, was unexpected due to the finding of no difference in oxygen utilisation between the two experiment conditions at the VL sites. Nevertheless, when considered in isolation, increased oxygen utilisation at the GN site during SIT, when compared to CMIE, is consistent with the increased power output, muscle fibre recruitment, and oxygen demand during high intensity exercise (Delp & Laughlin, 1998). No previous studies have published Δ[HHb] data from the GN when comparing SIT to CMIE. The GN muscle usually has a lesser role in power production during cycling than the larger VL muscles (Bijker, De Groot & Hollander, 2002). However, in an inactive cohort with no cycling specific training adaptation (no routine cycling activity was reported), it is possible that the smaller GN was recruited to a greater extent than in trained participants (Chapman et al., 2008), played an increased contributory role in power production during SIT and therefore exhibited an increased local oxygen utilisation when compared to CMIE. Additionally, the GN muscle has a greater percentage of oxidative muscle fibres (Hagström-Toft et al., 2002; Houmard et al., 1998) and greater citrate synthase activity than the VL muscles (Houmard et al., 1998). It would be expected that, under conditions demanding increased mechanical force production such as SIT, that a smaller muscle (GN) with a greater oxygen dependant muscle composition would have an increased oxygen utilisation response than larger muscles (LVL and RVL) with a greater glycolytic capacity (Laughlin & Roseguini, 2008).

The increased oxygen utilisation in the pre-frontal cortex during SIT, when compared to CMIE, is consistent with a previous comparison of these exercise modalities (Monroe et al., 2016). The current findings are also in agreement with a systematic review showing that exercise intensity is the only independent moderator of cerebral HHb, albeit during incremental exercise (Rooks et al., 2010). The higher Δ[HHb] in this area of the brain could be due to cerebral vasoconstriction, diminished cerebral blood flow, an increase in cerebral oxygen uptake and increased neuronal activity due to the higher exercise intensity and potentially the intensifying somatic cues of SIT (Bhambhani, Malik & Mookerjee, 2007; Curtelin et al., 2018; Ekkekakis, 2009; Mekari et al., 2015; Rooks et al., 2010; Smith & Billaut, 2010; Subudhi et al., 2008).

Whilst the beneficial effects of HIIT (and SIT) on metabolic and cardiovascular health parameters are well defined (Gibala et al., 2012; Gillen & Gibala, 2014, 2018), there is a distinct paucity of research examining the effects of HIIT on cerebral health parameters (Lucas et al., 2015). And whilst it must be acknowledged that oxygen utilisation in the pre-frontal cortex does not signify global cerebral oxygenation, cerebral perfusion or cortical activity, it is postulated that SIT represents a greater oxygen utilisation stimulus in the pre-frontal cortex, compared to CMIE, which may subsequently trigger positive cerebral adaptation (Lucas et al., 2015) and therefore signify one potential mechanism underlying the neuroprotective benefits of higher intensity exercise for cognitive and cerebrovascular health (Angevaren et al., 2007; Brown et al., 2012; Coetsee & Terblanche, 2017).

### Systemic oxygen consumption, heart rate and mechanical power

The significantly higher VO_2_, HR and mechanical power during SIT, when compared to CMIE, indicates an increased exercise intensity and integrated physiological demand during SIT. These findings are consistent with previous findings (Little et al., 2014; Olney et al., 2018) and were expected due to the study design (McKay, Paterson & Kowalchuk, 2009). The constant power output during CMIE, fixed at 50% of the peak power output achieved during MAX, produced physiological (VO_2_ and HR) responses consistent with moderate intensity exercise (Riebe et al., 2018).

### Blood pressure

Systolic blood pressure increased from rest to immediately post-exercise and then decreased to near resting levels during the 6 min recovery period in both conditions. No significant differences were found for SBP between SIT and CMIE.

Consistent with previous research (Rossow et al., 2010), no significant decrease in DBP occurred during the 6 min passive recovery period in both conditions, when compared to resting DBP. However, during the 6 min passive recovery period, a significantly lower DBP was observed during SIT when compared to CMIE at Rec 2, Rec 4 and Rec 6. This could possibly be due to peripheral vasodilation (and hence decrease in total peripheral resistance) (Goulopoulou, Fernhall & Kanaley, 2009; Hussain et al., 1996) and/or post-exercise peripheral hyperaemia in response to the SIT condition (Lacewell et al., 2014).

Some participants reported adverse sensations of dizziness and/or nausea (*n* = 7) during the recovery period of SIT. Symptoms were reported in the absence of statistically significant decreases in DBP during recovery, when compared to resting levels. This would suggest that the mechanism(s) causing these symptoms remain(s) to be elucidated. However, this assumption is made with caution, as a lack of a statistically significant decrease in group mean BP does not necessarily equate to no clinically significant effect on an individual basis, especially as the specific reduction in BP which triggers symptomatic PEH and its sequalae after high intensity exercise is unclear and probably highly individual (Halliwill et al., 2014). It is of interest to note that the mean reduction in DBP (resting DBP compared to lowest post-exercise DBP), whilst variable in both groups, was larger in the symptomatic individuals (mean = 11 mmHg) than in the asymptomatic individuals (mean = 7 mmHg). This finding leads authors to suggest that the effect of SIT *vs* CMIE on post-exercise BP, in participants with previous documentation of symptomatic PEH, may warrant future research.

### Physical activity enjoyment scale

The PACES is a reliable and valid measure of enjoyment during SIT and CMIE (Oliveira et al., 2013; Stork et al., 2017; Tritter et al., 2013). However, contradictory findings has led to a lack of clarity as to whether individuals find SIT or CMIE to be more enjoyable (Little et al., 2014; Olney et al., 2018; Stork, Gibala & Ginis, 2018; Thum et al., 2017; Williams et al., 2008). The lack of control for mechanical work, differences in protocol design and participant fitness/activity levels during previous research could explain the contradictory findings. Our finding of a higher level of enjoyment during CMIE, compared to SIT, represents a possible challenge with regards to SIT exercise prescription in a health context, as some of the positive health effects of HIIT may be due to the near maximal intensities achieved during higher intensity SIT protocols (Jimenez-Pavon & Lavie, 2017; Kessler, Sisson & Short, 2012). Participants reported adverse sensations of either leg muscle discomfort/cramping (*n* = 3) or dizziness and nausea (*n* = 7) during the recovery period of SIT. Although all participants felt well upon leaving the laboratory (no more than 30 min post-exercise), these adverse sensations could have contributed to the lower levels of enjoyment during SIT and highlight the importance of monitoring both physiological and perceptual indices during SIT interventions.

### Limitations

The physiological and perceptual responses during this study could have been influenced by the continuous *vs* intermittent nature of the CMIE and SIT conditions, respectively, obscuring the analysis of the variable: exercise intensity. It has been suggested that the intermittent format may be responsible for some of the positive physiological and perceptual responses during SIT and that intermittent moderate intensity exercise may provide similar positive responses without the potential compliance and safety issues of SIT (Jimenez-Pavon & Lavie, 2017). Therefore, the comparison of high and moderate intensity intermittent exercise formats, matched for mechanical work, is suggested as a future research direction.

To allow for the work-matched approach of this study, the CMIE condition was shorter in duration (range 5:33–7:38 min) than is routinely prescribed for health-related moderate intensity exercise interventions (10–30 min as per physical activity recommendations) (Riebe et al., 2018). Additionally, this study compared only one type of SIT to CMIE. It is therefore possible that this approach represents an external validity issue. However, time is the most frequently reported barrier to exercise participation and physical activity guidelines are increasingly incorporating phrases that acknowledge that any amount of physical activity is better than none for inactive individuals (and that the magnitude of benefit is inversely associated with the baseline activity level) (Blair, 2009; Egger et al., 2001; Myers et al., 2002). Therefore, the comparison of a 4 × 30 s SIT protocol to short-duration CMIE in this study provides practical and relevant information informing the selection of exercise format and intensity for inactive individuals wanting to initiate exercise in a time-efficient manner.

## Conclusions

In young inactive individuals, a session of SIT induced higher levels of oxygen utilisation, but lower levels of enjoyment than a work-matched session of CMIE. Furthermore, DBP was lower during the post-exercise period of SIT, compared to CMIE. The lower DBP during SIT coincided with pre-syncopal symptoms in some participants. In conclusion, these findings indicate that a higher level of physiological stress occurred during SIT which potentially contributed to lower post-exercise DBP and exercise enjoyment in this population, when compared to work-matched CMIE.

## Additional Information and Declarations

### Data Availability

The following information was supplied regarding data availability: The raw data is available at Figshare: Kriel, Yuri (2019): Yuri Kriel SIT vs CMIE. figshare. Dataset. DOI 10.6084/m9.figshare.7773968.v1.
